# Effects of Different Visual Color Stimuli on Stress Responses in Patients with Dental Phobia

**DOI:** 10.3390/jcm14196745

**Published:** 2025-09-24

**Authors:** Yukihiko Takemura, Kanta Kido, Toshiya Morozumi, Takuro Sanuki, Takeru Yao, Yoshiharu Mukai

**Affiliations:** 1 Department of Restorative Dentistry, Kanagawa Dental University, Yokosuka 2388580, Japan; takemura@kdu.ac.jp (Y.T.); mukai@kdu.ac.jp (Y.M.); 2 Department of Endodontics, The Nippon Dental University School of Life Dentistry at Niigata, Niigata 9518580, Japan; 3 Department of Dental Anesthesiology, Faculty of Dental Medicine and Graduate School of Dental Medicine, Hokkaido University, Sapporo 0608586, Japan; k.kido.hkd@gmail.com; 4 Department of Clinical Physiology, Institute of Biomedical Sciences, Nagasaki University, Nagasaki 8528523, Japan; sanuki-t@nagasaki-u.ac.jp; 5 Tottori Prefectural Central Hospital, Tottori 6800901, Japan; take808099@gmail.com

**Keywords:** anxiety, pain, salivary alpha-amylase, dental phobia, color stimulation, visual analog scale

## Abstract

**Background:** Dental phobia significantly impairs treatment adherence and oral health–related quality of life. While intravenous sedation (IVS) is commonly used to manage anxiety, interest in non-pharmacological alternatives is increasing. Visual color stimuli are known to affect emotional processing and autonomic nervous system activity. This study investigated whether brief exposure to colored goggles (translucent, green, blue, red) influenced physiological and psychological stress markers in patients with dental phobia undergoing IVS. **Methods:** Twenty patients with dental phobia (CMDAS ≥ 20) participated in a within-subject repeated measures design, experiencing all four color conditions in randomized order via Latin square. Salivary alpha-amylase (sAA), heart rate (HR), and Visual Analog Scale (VAS) scores for pain and satisfaction were measured at four time points. The Friedman test, followed by Wilcoxon signed-rank tests with Bonferroni correction, was used to compare conditions. **Results:** Prior to peripheral intravenous cannulation (PIC), green goggle exposure significantly reduced median sAA levels compared to the translucent control (*p* = 0.009). HR was lower in the green and blue conditions than in the red, although not significantly. VAS pain scores were significantly reduced under green and blue conditions; satisfaction scores remained uniformly high across all conditions. Red did not worsen stress responses compared to the control and may promote increased engagement. **Conclusions:** Cool color stimuli—particularly green and blue—may attenuate acute stress responses in patients with dental phobia prior to PIC. Incorporating such visual cues into preoperative environments may serve as a simple, non-invasive adjunct to managing dental anxiety.

## 1. Introduction

Dental phobia presents a significant clinical challenge, often leading to treatment avoidance and discontinuation of dental care. This condition not only contributes to the deterioration of oral health but is also closely associated with a reduced quality of life, underscoring its clinical relevance.

The development of dental phobia is widely recognized as multifactorial. Contributing factors include previous painful dental experiences [1], psychological predispositions such as heightened anxiety sensitivity [2] and sympathetic nervous system hyperreactivity [3], as well as social influences like family dynamics [4] and educational background [5]. In addition, the dental clinical environment itself is a major source of stress for many patients [6]. Situations involving unpredictability, lack of control, and poor visual feedback [7], as well as multisensory stimuli—such as noise, vibration, lighting, and chemical odors—are known to provoke anxiety and fear [8]. These stressors are thought to activate the sympathetic–adrenal–medullary (SAM) system [9], eliciting acute aversive responses. Under threatening conditions, both the SAM and hypothalamic–pituitary–adrenal (HPA) axes may be simultaneously activated, leading to systemic stress responses—such as increased heart rate (HR) and perspiration—via catecholamine release [10]. While the HPA axis primarily regulates chronic stress through cortisol secretion [11], salivary alpha-amylase (sAA) has emerged as a reliable biomarker of acute stress, reflecting SAM activity [9]. sAA levels rise rapidly within minutes of acute stress exposure, making it a practical and non-invasive measure for real-time stress evaluation, particularly in dental settings.

In clinical dentistry, intravenous sedation (IVS) with benzodiazepine-based anxiolytics is frequently employed to manage dental anxiety [12]. However, concerns regarding side effects—including dependence, delirium, and respiratory depression—limit its long-term or repeated application [13]. Consequently, there is increasing interest in non-pharmacological and non-invasive strategies to alleviate stress and anxiety in dental patients.

Among various environmental elements in dental settings, visual stimuli have been shown to significantly influence both psychological and physiological responses [14]. In particular, color-based visual stimuli are known to modulate emotional processing and autonomic nervous system activity [15]. Beyond serving as visual cues, color signals received by the retina are transmitted not only to the visual cortex but also to emotional and autonomic control centers such as the amygdala and hypothalamus [16]. Through these visual–emotional pathways, color stimuli can influence the balance between sympathetic and parasympathetic activity, thereby affecting arousal, emotional states (e.g., anxiety, fear), attentional focus, and behavioral responses [17]. Retinal input to the suprachiasmatic nucleus (SCN) has also been shown to suppress melatonin secretion and activate serotonergic systems, leading to downstream effects on biological rhythms and endocrine function [18].

Different hues have been reported to induce distinct psychological and physiological responses [19]. Specifically, cool colors such as green and blue are often associated with calming effects. Green, frequently linked to natural environments, has been reported to evoke positive emotions such as reassurance and tranquility [20]. Meanwhile, blue has been shown to influence autonomic regulation via the brainstem and limbic system, promoting a parasympathetic-dominant state [21]. Conversely, red—a warm color—is typically associated with heightened arousal and increased psychological tension [21], and is generally avoided in stress-reducing environments. Nevertheless, red has also been reported to enhance motivation and promote proactive behavior [22], suggesting that its effects may be context-dependent—even potentially beneficial in certain clinical situations.

Based on these findings, this study aimed to examine the effects of visual color stimuli—delivered through goggles with translucent, green, blue, or red lenses—on stress responses in patients with dental phobia during dental procedures under IVS.

## 2. Materials and Methods

### 2.1. Study Design

This study employed a within-subject repeated measures design to evaluate the effects of color-based visual stimuli on physiological and psychological stress responses in patients with dental phobia. To control for interindividual variability, each participant was exposed to four color conditions—translucent, green, blue, and red—on separate days. The order of exposure was determined using a Latin square design and randomly assigned to minimize order and carryover effects.

Participants were blinded to the assigned color condition in each session, and outcome assessors were arranged independently to prevent bias from the color condition.

This study was conducted as a prospective observational study. It was performed in accordance with the Declaration of Helsinki and approved by the Ethics Committee of Kanagawa Dental University (Approval Nos.: 554, 601, 615, and 937). The study was registered in the UMIN Clinical Trials Registry (UMIN000038122). Written informed consent was obtained from all participants.

### 2.2. Participants

Twenty patients (9 males, 11 females) who underwent dental treatment under IVS at Kanagawa Dental University Hospital between January 2019 and December 2024 were enrolled in this study. All participants met the diagnostic criteria for dental phobia, as assessed by the Customized Modified Dental Anxiety Scale (CMDAS) (Table 1).

The CMDAS is based on the Modified Dental Anxiety Scale [23] and consists of five items (Table 2), each rated on a five-point Likert scale from 1 (“not anxious at all”) to 5 (“extremely anxious”), yielding a total score ranging from 5 to 25.

Inclusion criteria were as follows: (1) a CMDAS score of ≥20 and (2) the presence of severe anxiety-related behaviors during the initial consultation that interfered with treatment—such as strong treatment refusal, physical rigidity, marked increases in blood pressure or heart rate, or voluntary discontinuation of treatment. Patients who met only one of these criteria were excluded.

The cutoff of CMDAS ≥ 20 was based on preliminary clinical observations: all patients with scores ≥ 20 required IVS due to severe anxiety-related behaviors, whereas some with scores below 19 could be managed with non-pharmacological approaches. Therefore, a threshold of 20 was considered appropriate. Moreover, the CMDAS is a customized version of the MDAS, modified to fit our institution’s clinical environment, with standardized questions to minimize bias. Since the MDAS cutoff of ≥19 is internationally recognized as indicating high dental anxiety, our threshold is consistent with international standards.

In addition, participants’ medical and psychological histories were reviewed, and none had previous experience with chromotherapy or specific relaxation techniques. Thus, these factors were excluded as potential confounders.

All participants were between 18 and 79 years of age, cognitively intact, and capable of completing self-administered questionnaires. Written informed consent was obtained from all participants.

Patients with systemic diseases or those taking medications that could influence salivary flow or sAA activity were excluded.

### 2.3. Visual Stimuli and Controlled Environment

Four color conditions were used as visual stimuli: translucent, green (530–547 nm), blue (466–482 nm), and red (631–648 nm). After the pre-treatment interview, participants wore goggles corresponding to the assigned color condition, which were kept on throughout the entire dental procedure. To eliminate anticipatory bias, participants were blinded to the color they would receive in each session.

Peripheral intravenous cannulation (PIC) was standardized to occur at least 20 min after goggle application. All participants completed all four color conditions in a crossover manner, with the presentation order determined by a Latin square design.

**Figure 1:**
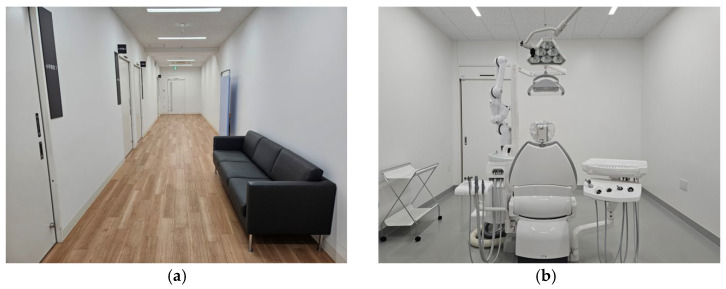
Standardized clinical environment. (**a**) Waiting room, (**b**) Treatment room. Walls were uniformly white, and natural light was eliminated to control environmental factors.

The dental operatory environment was standardized across all sessions, including lighting (neutral white fluorescent), room temperature (25–27 °C), dental instruments, operator, and ambient sound (silence maintained). The walls of both the waiting and treatment rooms were uniformly white, and all windows were blocked to eliminate natural light interference (Figure 1).

The same type of dental treatment or instruments was not strictly applied across all patients, nor were identical procedures repeated under each color condition. However, all patients attended at least four visits and received dental treatment under each of the four color conditions.

### 2.4. Outcome Measures and Measurement Timing

**Figure 2:**
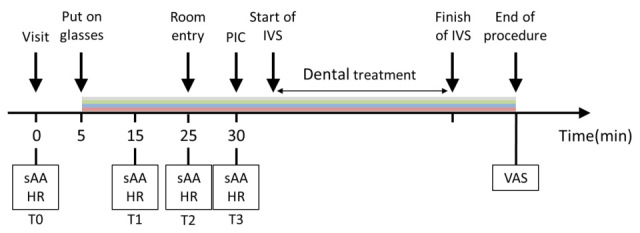
Study protocol and time points for measurement. sAA: salivary alpha-amylase, HR: heart rate, PIC: peripheral intravenous cannulation, VAS: visual analog scale, IVS: intravenous sedation. T0: Upon being seated in the waiting room, T1: 10 min after wearing the goggles, T2: Immediately after entering the treatment room, T3: Just before PIC.

To comprehensively assess stress responses, both physiological and psychological indicators were evaluated. Physiological responses included sAA activity and HR, while psychological responses were assessed using visual analog scale (VAS) scores for pain and overall satisfaction. Patients wore the goggles from the waiting room and continued to wear them during and after the treatment until the VAS assessment was completed (Figure 2).

Measurements were taken at four predefined time points:

T0: Upon being seated in the waiting room (baseline);

T1: Ten minutes after goggle application (isolated effect of visual stimuli);

T2: Immediately after entering the treatment room (response to environmental change);

T3: Just before PIC (anticipated peak stress).

#### 2.4.1. Measurement of sAA Activity

sAA activity was measured using a dry clinical chemistry analyzer, the Salivary Amylase Monitor (NIPRO Corporation, Osaka, Japan) [24]. Unstimulated saliva was used for the measurement, and participants were instructed to refrain from eating, drinking, smoking, and exercising for at least 3 h before sample collection. The procedure was conducted under resting conditions following standardized protocols. sAA was regarded as an immediate physiological stress marker that sensitively reflects sympathetic nervous system activity.

#### 2.4.2. Measurement of HR

HR was measured at rest using an automated clinical blood pressure and pulse monitor (Dental Moneo BP-A308D, Fukuda Denshi Co., Ltd., Tokyo, Japan). To minimize the influence of conversation and physical movement, participants were kept at rest immediately before the measurement.

#### 2.4.3. Measurement of VAS

After the completion of dental treatment, participants were asked to provide subjective evaluations using VAS for two items: “pain during peripheral intravenous cannulation” and “overall satisfaction with dental treatment.”

### 2.5. Statistical Analysis

To compare sAA activity, HR, and VAS scores across the four visual conditions, the Friedman test was applied to repeated measures data. Where significant main effects were observed, post hoc pairwise comparisons were conducted using the Wilcoxon signed-rank test. Bonferroni correction was applied to adjust for multiple comparisons.

All statistical analyses were performed using EZR (version 1.54; Saitama Medical Center, Jichi Medical University, Saitama, Japan). Data are presented as median (IQR) for continuous variables and as n (%) for categorical variables.

The sample size was determined using G*Power (version 3.1.9.2; Heinrich-Heine-Universität Düsseldorf, Düsseldorf, Germany), based on a moderate effect size (Cohen’s d = 0.5), a significance level of 0.05, and a power of 0.8 for repeated measures analysis. Accordingly, a target of 20 participants was set. In addition, the feasibility of case accumulation and ethical considerations were taken into account in determining the final sample size.

## 3. Results

The results are presented as medians (interquartile ranges).

### 3.1. sAA Activity Measurement

**Figure 3:**
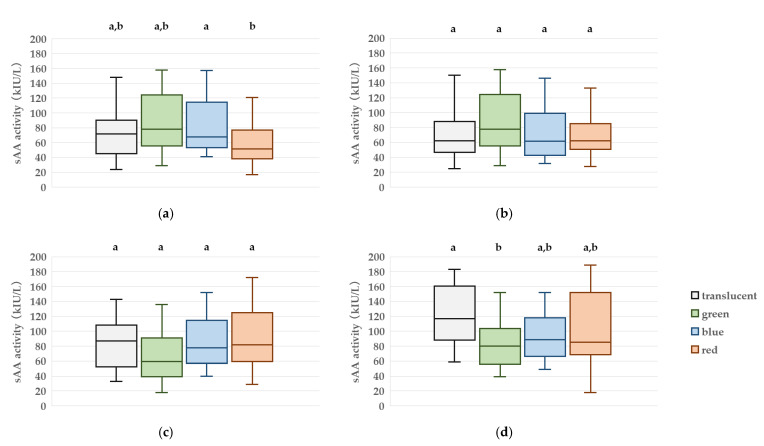
Box plots of salivary alpha-amylase (sAA) activity at four time points: (**a**) T0 (baseline in the waiting room), (**b**) T1 (10 min after goggle application), (**c**) T2 (immediately after entering the treatment room), (**d**) T3 (just before peripheral intravenous cannulation). Boxes represent the interquartile range (IQR), the horizontal line within each box indicates the median, and the whiskers show the minimum and maximum values. Different letters denote statistically significant differences between groups (*p* < 0.05, n = 20).

At T3, a significant difference in sAA activity was observed among the visual color conditions. The median (IQR) sAA values were 80.0 (61.3–100.5) kIU/L for the green condition, 88.5 (67.5–112.8) kIU/L for blue, 88.5 (70.3–149.3) kIU/L for red, and 117.0 (90.8–160.3) kIU/L for the translucent condition. Post hoc pairwise comparisons indicated that the green condition produced significantly lower sAA activity than the translucent condition (*p* = 0.009). Although the blue condition exhibited a trend toward lower values compared to the translucent condition, this did not reach statistical significance. No significant differences were found among the green, blue, and red conditions (Figure 3).

### 3.2. HR Measurement

**Figure 4:**
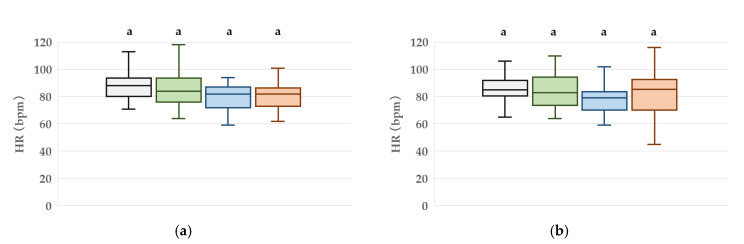

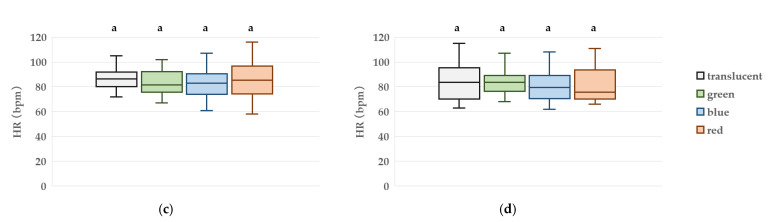
Box plots of heart rate (HR) at four time points: (**a**) T0 (baseline in the waiting room), (**b**) T1 (10 min after goggle application), (**c**) T2 (immediately after entering the treatment room), (**d**) T3 (just before peripheral intravenous cannulation). Boxes represent the interquartile range (IQR), the horizontal line within each box indicates the median, and the whiskers show the minimum and maximum values. Different letters denote statistically significant differences between groups (*p* < 0.05, n = 20).

At T3, the median HR was 83.5 (76.8–89.0) bpm for the green condition, 79.5 (71.5–89.0) bpm for blue, 75.5 (70.8–93.3) bpm for red, and 83.5 (72.8–94.5) bpm for the translucent condition. No statistically significant differences in HR were observed among the four conditions (Figure 4).

### 3.3. VAS Measurement

#### 3.3.1. Pain During PIC

At T3, the median VAS scores for pain were 18.0 (3.5–68.3) for the green condition, 11.0 (5.3–19.0) for blue, 34.5 (17.5–68.8) for red, and 59.5 (36.3–84.0) for the translucent condition. Multiple comparisons showed that pain scores were significantly lower in the green condition compared to red (*p* = 0.041) and translucent (*p* = 0.046), and significantly lower in the blue condition compared to red (*p* = 0.014) and translucent (*p* = 0.014). No significant difference was observed between the green and blue conditions.

#### 3.3.2. Satisfaction with the Treatment

**Figure 5:**
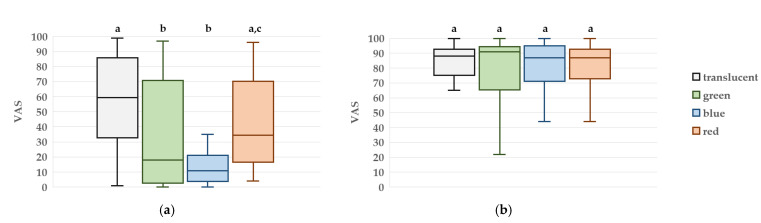
Box plots of visual analog scale (VAS) scores at four time points: (**a**) pain during peripheral intravenous cannulation, (**b**) overall satisfaction with dental treatment. Boxes represent the interquartile range (IQR), the horizontal line within each box indicates the median, and the whiskers show the minimum and maximum values. Different letters denote statistically significant differences between groups (*p* < 0.05, n = 20).

The median VAS scores for overall treatment satisfaction were 91.0 (69.8–93.5) for the green condition, 87.0 (73.8–95.0) for blue, 87.0 (74.3–92.3) for red, and 88.0 (77.0–92.3) for the translucent condition. No statistically significant differences were observed among the four conditions (Figure 5).

## 4. Discussion

This study investigated the effects of chromatic visual stimulation on stress responses in patients with dental phobia, using both physiological and psychological indicators. Among the four visual conditions—translucent (control), green, blue, and red—green stimulation significantly reduced both sAA activity and VAS scores, suggesting a clear stress-reducing effect across both physiological and psychological domains. In contrast, blue stimulation significantly reduced VAS scores but did not affect sAA activity. Red stimulation showed no significant improvements in either indicator and elicited stress responses comparable to or greater than those under the control condition. No significant differences in HR were observed among the color conditions, suggesting that short-term visual stimuli may have a limited influence on HR. HR is a general indicator of autonomic nervous system activity, but is susceptible to individual variability and external factors, which makes it less sensitive to immediate stress responses compared with sAA. In addition, the effects of IVS may have further suppressed fluctuations in HR, which could explain the absence of statistically significant differences.

Green is widely recognized as a color that evokes calmness and safety due to its association with natural environments and is known to promote parasympathetic nervous system activity [20]. Exposure to green light has been associated with relaxation-related indicators such as stabilized heart rate, reduced respiratory rate, and increased alpha wave activity on the EEG. Our previous study also demonstrated that green light stimulation prior to dental procedures significantly reduced sAA activity [25], which is consistent with the present findings. Moreover, animal studies have shown that exposure to green LED light activates descending pain inhibitory pathways involving the brainstem and spinal cord, leading to increased levels of endogenous opioids such as enkephalins, thereby producing analgesic effects [26]. These effects were blocked by naloxone, indicating that the analgesic mechanism of green light stimulation involves opioid-mediated pathways. In addition, green light has been suggested to suppress amygdala hyperactivity and enhance emotional regulation through the amygdala–prefrontal cortex network [27]. Taken together, these findings suggest that green visual stimulation contributes to anxiety reduction and increased pain thresholds via central nervous system mechanisms, thus exerting stress-relieving effects on both physiological and psychological levels.

Blue stimulation resulted in significantly lower VAS scores but did not significantly reduce sAA activity. As a prototypical cool color, blue has been reported to induce emotional calmness and activate parasympathetic responses via the brainstem and limbic structures [21]. Environmental exposure to blue light has been linked to reductions in blood pressure and heart rate, as well as suppression of sweat secretion, suggesting a potential for temporary anxiety relief. Consistent with these findings, our study showed that blue stimulation produced significantly lower VAS scores compared to red and translucent conditions, indicating a psychological calming effect. However, blue light also exhibits physiological arousal properties [28]. Short-wavelength blue light activates melanopsin-containing retinal ganglion cells, which stimulate SCN, suppress melatonin secretion, and enhance sympathetic activity. This arousal effect may have counteracted any potential reduction in sAA activity, resulting in the observed discrepancy between subjective and physiological responses. Furthermore, the stress-relieving effects of blue stimulation appear less consistent than those of green. For example, in patients with fibromyalgia, green light significantly reduced anxiety, whereas blue light did not [29]. These findings suggest that under blue light, subjective perceptions of calmness may not always be accompanied by measurable physiological changes, highlighting a potential dissociation between psychological and physiological indicators. Therefore, a comprehensive evaluation of stress-relieving interventions should include both subjective (e.g., self-reported anxiety) and objective (e.g., biomarkers) assessments. Ideally, both should align to demonstrate consistent calming effects; however, even in the absence of physiological changes, the psychological comfort induced by blue stimulation may still enhance patient tolerance and cooperation during dental treatment.

Red stimulation did not significantly reduce either subjective anxiety or physiological stress. Red is known to activate the sympathetic nervous system, leading to increases in heart rate, blood pressure, and salivary catecholamine levels [21]. Accordingly, it is typically associated with heightened emotional arousal and vigilance. For patients with dental phobia—who are already experiencing elevated psychological stress—exposure to red light may further amplify anxiety and tension. The present findings align with this interpretation. Conversely, red has also been reported to enhance attention, improve concentration, and promote active coping strategies and cognitive reappraisal [22]. Under certain conditions, these effects may facilitate greater engagement and adaptive behavior during dental treatment. However, these potential benefits are highly context-dependent and may vary according to the patient’s psychological readiness and baseline arousal level. In individuals with high anxiety, the arousing properties of red may be counterproductive, exacerbating stress rather than alleviating it.

Therefore, while red stimulation did not demonstrate stress-reducing effects in this study, its potential to facilitate adaptive behavior under specific clinical conditions warrants further investigation.

No significant differences in overall patient satisfaction with dental treatment—as measured by VAS scores—were observed among the color conditions. This result likely reflects the strong sedative effects of IVS, which may have elevated satisfaction levels uniformly across all groups [30].

This study has several limitations. First, the relatively small sample size inevitably restricts the statistical power and generalizability of the findings. Second, sAA was the only physiological marker assessed; stress-related biomarkers from the HPA axis, such as cortisol, were not evaluated. Future studies should include HPA-related markers to enable a more comprehensive assessment of stress responses. Third, the color stimulation used in this study was brief and transient; thus, the observed effects may be limited to short-term exposure. Finally, the study was conducted at a single institution under tightly controlled conditions, including a standardized procedure (peripheral intravenous cannulation) performed by the same operator. Therefore, the generalizability of these findings to other clinical settings, treatment types, or patient populations should be interpreted with caution. To enhance clinical applicability, future studies should consider multicenter designs, longer exposure durations, and more diverse patient populations.

In conclusion, chromatic visual stimulation appears to be a simple and non-invasive strategy for alleviating both physiological and psychological stress in patients with dental phobia. In particular, green stimulation may suppress excessive sympathetic activity and promote a sense of calm, making it a potentially useful adjunct in clinical practice. Incorporating color design into dental environments may contribute to improving patient quality of life and optimizing treatment conditions.

## 5. Conclusions

This study demonstrated that chromatic visual stimuli exert differential effects on both physiological and psychological stress responses in patients with dental phobia. In particular, green stimulation significantly reduced both types of responses, indicating its consistent stress-relieving potential as a non-pharmacological adjunct in dental clinical practice. Color stimuli are low-cost and non-invasive, and may serve as a complementary approach to IVS, particularly valuable for patients at high risk or with heightened sensitivity to pharmacological interventions. In addition, while the present findings highlight short-term effects, future studies should evaluate the potential clinical utility of longer-term or continuous exposure to color stimuli.

## Figures and Tables

**Table 1:** Demographic characteristics of study participants. Continuous variables are reported as median (IQR).

Patient Characteristics	Total (n = 20)
Continuous Variables	Median (IQR)
Age (years)	42.6 (36.2–48.2)
Height (cm)	165.3 (158.8–169.7)
Weight (kg)	64.8 (56.8–71.1)
Body Mass Index (BMI)	22.8 (19.0–25.1)
**Categorical Variables**	
Sex [Male/Female]	9/11
Presence of Color Vision Deficiency	0

**Table 2:** Items of the Customized Modified Dental Anxiety Scale (CMDAS). Each item is rated on a five-point Likert scale (1 = not anxious at all, 5 = extremely anxious), with total scores ranging from 5 to 25.

Items
(1) Do you feel anxious when making a dental appointment?
(2) Do you feel anxious while waiting in the reception area before treatment begins?
(3) Do you feel anxious when instruments are inserted into your mouth?
(4) Do you feel anxious about surgical dental procedures?
(5) Do you feel anxious about receiving an anesthetic injection in your mouth?

## Data Availability

The data presented in this study are available from the corresponding author upon reasonable request. The data are not publicly available due to ethical restrictions.
